# Assessment of Combined Food and Nutrition Insecurity Screening in the Emergency Department: A Brief Report

**DOI:** 10.5811/westjem.64320

**Published:** 2026-10-13

**Authors:** Marco Santos Esteban, Jolie Minh-Trang Nguyen, Ian Dennis Capo Olliffe, Bharath Chakravarthy, Victor Cisneros

**Affiliations:** * University of California, Irvine, Department of Emergency Medicine, Irvine, California; † University of California San Diego School of Medicine, San Diego, California; ‡ Eisenhower Health, Department of Emergency Medicine, Rancho Mirage, California

## Abstract

**Introduction:**

Emergency department (ED) food insecurity (FI) screening traditionally identifies insufficient access to food but does not account for diet quality. We evaluated the incremental yield of adding a nutrition insecurity (NI) screener to routine FI screening.

**Methods:**

This single-center observational study enrolled adult ED patients from April–September 2025. Participants completed the 2-item Hunger Vital Sign and a 1-item NI screener. Our primary measure was incremental yield of NI screening beyond FI screening, defined by the number of NI-positive/FI-negative patients identified. Secondary outcome measures included FI and NI prevalence, screening concordance, and number needed to screen. Screening fidelity was supported through structured training of research associates and standardized phrasing.

**Results:**

Of 1,120 patients, 13.6% (95% CI, 11.6–15.7%) screened positive for FI and 11.4% (95% CI, 9.7–13.5%) for NI. While the measures showed substantial agreement (κ = 0.67), they identified significantly different populations (McNemar *P* = .01). Specifically, 28 patients (2.5%) screened NI-positive but FI-negative, yielding an 18.4% relative increase in identified at-risk patients compared to only screening for FI.

**Conclusion:**

Dual screening identified a clinically relevant NI-only subgroup whose diet-quality barriers were not captured by FI screening alone. Incorporating NI into ED social needs assessments may improve identification of diet-related risk and guide medically tailored interventions.

## INTRODUCTION

Diet-sensitive chronic diseases frequently precipitate emergency department (ED) visits, yet routine food insecurity (FI) screening using tools such as the 2-item Hunger Vital Sign primarily captures limited or uncertain access to sufficient food rather than diet quality.^1^,^2^ Nutrition insecurity (NI) screening extends beyond traditional FI screening to assess nutritional quality, capturing barriers to accessing healthy, nutrient-dense foods.^2^ Previous community-based screenings have suggested that using both FI and NI screeners can identify individuals at highest risk for poor diet and health outcomes better than either alone.^3^ However, the incremental value of combined FI+NI screening in the ED remains unexamined.

We implemented a dual FI+NI screening workflow to estimate the prevalence of FI and NI, and to quantify the incremental yield of NI screening among FI-negative patients. We hypothesized that adding a brief NI screener would identify a distinct NI-only subgroup not captured by FI alone, consistent with prior evidence outside the ED.^3^ Identifying NI would serve to address diet-sensitive chronic conditions commonly driving ED visits through better informed ED discharge counseling, referral decisions, and targeted linkage to “food is medicine” interventions for patients.^4^ Produce prescriptions, which address barriers to food quality rather than quantity, represent one such intervention; a multisite evaluation of nine programs in low-income communities demonstrated clinically meaningful improvements in glycated hemoglobin, blood pressure, and body mass index among adults with poor cardiometabolic health, underscoring the patient-oriented benefit of identifying the NI-only subgroup that FI screening alone would miss.^5^

## METHODS

The primary research question was whether adding a brief NI screening item to standard FI screening would identify an additional subgroup of ED patients with diet-quality barriers who would not be detected by FI screening alone.

### Screening

This single-center, observational cross-sectional study was conducted at a large, urban, academic emergency department with approval from the University of California, Irvine Institutional Review Board (Protocol #2017-4029). Research associates (RA) were scheduled in 4-hour shifts from 8:00 am to 12:00 am daily from April to September 2025, during which all data were collected. Participants were recruited using a convenience sampling strategy during RA coverage hours. Eligible participants were adults (18 years of age and older) who spoke English or Spanish. For Spanish-speaking participants, bilingual RAs provided oral translation and delivery of questionnaire items. Exclusion criteria included individuals from protected populations (pregnant, psychiatric emergency, or incarcerated) and those who spoke languages other than English or Spanish.

Screenings for participant eligibility were conducted by RAs after triage using the secure electronic health record (EHR) (Epic Systems Corporation) platform. After confirming eligibility, RAs approached patients at their convenience and without delaying clinical care to administer a dual screening survey. The survey consisted of the two-item Hunger Vital Sign questionnaire to screen for FI and a single preliminary NI screening question from the Household Nutrition Security measure developed by Calloway et al.^1^,^6^ The Household Nutrition Security measure was selected for its brevity compared with alternative nutrition security measures. Specifically, the N1 question was selected as the screening question: “In the last 12 months, (I/we) had to eat some foods that were not good for my health and well-being because (I/we) couldn’t get other types of food.” Participants who responded “sometimes,” “often,” or “always” on a standardized 5-point Likert frequency scale ranging from “Never” to “Always” were registered as experiencing NI. All RAs used standardized scripts when delivering the survey with periodic fidelity checks.

Individuals identified as experiencing FI and/or NI were provided a printed food assistance guide detailing local food pantries and supplemental community resources.

**Population Health Research Capsule**

What do we already know about this issue?

*Food insecurity screening identifies limited access to food but may miss diet-quality barriers. Nutrition insecurity screening is a promising tool to address this.*

What was the research question?

*Does nutrition insecurity screening identify a meaningful population of emergency department patients missed by food insecurity screening?*

What was the major finding of the study?

*Nutrition insecurity screening identified a meaningful separate population of patients missed by food insecurity screening (2.5%; 95% CI, 1.7–3.6%); McNemar χ* *^2^* *= 6.61; P = .01.*

How does this improve population health?

*Dual screening identifies additional patients with diet-quality barriers, enabling targeted nutrition interventions and improved health equity.*

### Statistics

We calculated prevalence estimates for FI and NI with 95% CIs. Participants were stratified into four mutually exclusive groups: FI-only, NI-only, concurrent FI and NI, or neither. Screening concordance was assessed using the Cohen kappa coefficient. We used the McNemar chi-squared test to analyze discordant cases (FI-only vs NI-only) to determine whether the two tools identified significantly different unique populations.

The incremental yield of NI screening was defined as the percentage increase in at-risk individuals identified beyond the baseline FI-positive group (NI-only cases divided by total FI-positive cases). The number needed to screen to determine one new at-risk patient was calculated as the total sample size divided by the number of NI-only cases. Statistical significance was set at *P* < .05.

Eligible adults (18 years of age or older) who spoke English or Spanish were screened using the Hunger Vital Sign FI and a 1-item NI question. Patients were classified as FI and/or NI if they provided any positive response to the FI items or selected “always,” “sometimes,” or “often” for the NI item, and they were subsequently eligible for study enrollment. All other participants were considered food/nutrition secure and excluded from enrollment, study of ED-based dual screening for food and nutrition insecurity among adult patients.

## RESULTS

Of 1,120 screened patients, mean age was 51.7 (21.9) years and 47.4% were female. Of these patients, 152 participants (13.6%; 95% CI, 11.6–15.7%) screened positive for FI and 128 (11.4%; 95% CI, 9.7–13.5%) for NI (Table). While the two measures showed substantial agreement (Cohen κ = 0.67; 95% CI, 0.61–0.74), the McNemar test confirmed they identified significantly different populations (McNemar χ^2^ = 6.61, *P* = .01). Specifically, while 52 patients (4.6%) were identified only by the FI screener, 28 patients (2.5%) were identified only by the NI screener. These 28 NI-only patients represent an 18.4% incremental yield to detect at-risk individuals who would have been missed by standard FI screening alone. The number needed to screen to identify one such unique NI-positive patient was 40.

## DISCUSSION

This study demonstrates that a combined FI and NI screening protocol in the ED identifies a meaningful population of at-risk individuals who would be missed by FI screening alone. Among 1,120 patients screened in the ED, 13.6% screened positive for food insecurity, and 11.4% for nutrition insecurity. As expected due to the intertwined nature of FI and NI, substantial agreement (κ = 0.67) was observed between the measures. However, the McNemar test *(P* = .01) confirmed that the NI screener identifies a distinct clinical subgroup. Consequently, the 28 patients (2.5%) identified exclusively by NI screening represent a meaningful 18.4% incremental yield in detecting at-risk patients beyond traditional food insecurity screening.

This NI-only subgroup includes patients who reported having enough food but still face structural barriers to nutritious options, such as living in low-access food areas or being unable to afford fresh produce.^7^, ^8^ These barriers can limit diet quality and make it more difficult to manage diet-sensitive chronic disease.^9^ Poor diet quality is estimated to contribute to 45% of US cardiometabolic deaths and is a primary driver of conditions including diabetes, hypertension, and heart failure—conditions that commonly precipitate ED visits and are worsened by dietary nonadherence.^10^ Nutritional insecurity screening therefore has direct ED relevance because identifying this subgroup opens a pathway to tailored interventions, such as produce prescriptions, which bypass logistical and financial hurdles and have been demonstrated to lead to improved health status and clinically related outcomes, as seen in an evaluation including adults with poor cardiometabolic health.^5^ Without such targeted screening, these structural barriers remain undetected, missing an opportunity for personalized linkage to care.[Fig f1-wjem-27-1684]

This novel and preliminary study provides a baseline for the utility of dual FI and NI screening in the ED using a simple screening tool. However, further work is necessary to explore the feasibility of NI in this setting and an optimized intervention. In routine ED practice, NI screening would likely be most actionable if incorporated into existing intake, triage, or social-needs screening workflows and documented in a structured EHR field. A positive NI screen could trigger a visible chart flag, best-practice advisory, or discharge-planning prompt that links clinicians to standardized response options, such as social work consultation, printed after-visit resources, community food program referrals, produce prescription programs, or medically tailored grocery programs.^11^,^12^ To minimize clinician burden, these pathways should rely on structured documentation, automated resource generation, and clear referral criteria rather than requiring clinicians to independently identify and address each positive screen.

Additionally, further demographic characterization of this subgroup is essential for health equity. While racial and ethnic disparities in food insecurity are well-established, it remains unknown whether NI follows similar patterns or affects distinct demographic groups facing unique barriers.^13^ Clarifying these associations will allow health systems to implement culturally sensitive interventions. Furthermore, given recent instability in federal food assistance, such as the Supplemental Nutrition Assistance Program and the Local Food Assistance Program, and the resulting strain on vulnerable communities, establishing efficient ED screening protocols is urgent to ensure at-risk patients are identified and linked to stabilizing resources.^14^, ^15^

## LIMITATIONS

This study has several limitations. First, the reliance on convenience sampling and restriction to English and Spanish speakers introduces selection bias, in addition to social desirability bias due to RA-administered surveys. Additionally, the RAs did not track the total number of eligible patients who were not approached, limiting our ability to report the proportion of all ED patients surveyed for FI and NI. The study’s scope was also limited to adults (18 years of age or older). Together, these methodological constraints limit the generalizability of our findings to the broader ED population and may have influenced FI and NI estimates by under-representing patients who presented outside enrollment hours, were missed during high-volume periods, or faced language barriers. Second, we used item N1 (“In the last 12 months, we had to eat some foods that were not good for our health...”) rather than the recommended screener item N3.

In the original development study, item N3 was specifically selected for its high sensitivity (93%) in identifying at-risk households. However, item N1 was selected since it was a stronger indicator of the Household Nutrition Security construct and its wording directly captures access-related barriers to consuming healthful foods more closely than item N3.^6^ It should be noted that item N1 is not validated in an ED setting, an important limitation. Additionally, while using N1 prioritized construct validity in NI screening, it was not optimized for maximum screening sensitivity, suggesting that our reported prevalence (11.4%) may represent a conservative estimate of the true burden of nutrition insecurity.^3^, ^5^

Further, the variability in administration format (tablet vs verbal) may have influenced response accuracy; specifically, the reduced privacy of verbal screening compared to self-administration may have elicited social desirability bias, potentially leading to the underreporting of both food and nutrition insecurity.^16^ Lastly, no downstream clinical outcomes or resource utilization were examined following screening.

## CONCLUSION

This preliminary study demonstrates the added benefit of a dual FI+NI screening protocol in the emergency department, which identified a subset of patients struggling only with nutritional insecurity. Beyond expanded detection, NI screening distinguishes between limitations in food quantity and deficits in diet quality. This differentiation presents an opportunity to provide better-tailored medical interventions focused on food quality, such as produce prescriptions, to a targeted patient population in the ED. Future efforts should prioritize integrating this brief screener into the electronic health record and pairing it with appropriate intervention and follow-up.

## Figures and Tables

**Figure f1-wjem-27-1684:**
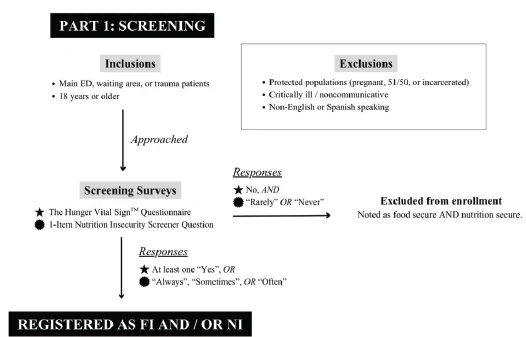
Flowchart of emergency department dual screening protocol in a study evaluating the incremental yield of adding a nutrition insecurity screener to routine food insecurity screening. *ED*, emergency department; *51/50*, Section 5150 of the California Welfare and Institutions Code allowing a clinician to place a person on an involuntary 72-hour psychiatric hold.

**Table:** Summary of screening outcomes for food insecurity and nutrition insecurity among emergency department patients (*N* = 1,120).

Screening Outcome	*n* (%)	95% Confidence Interval
Food insecurity (FI) positive	152 (13.6%)	11.6–15.7
Nutrition insecurity (NI) positive	128 (11.4%)	9.7–13.5
FI only	52 (4.6%)	3.5–6.1
NI only	28 (2.5%)	1.7–3.6
Both FI and NI	100 (8.9%)	7.4–10.8
Neither FI nor NI	940 (83.9%)	81.6–86.0

κ = 0.67 (95% CI, 0.61–0.74); McNemar χ^2^ = 6.61, P = .01.
